# cGAS-STING signaling encourages immune cell overcoming of fibroblast barricades in pancreatic cancer

**DOI:** 10.1038/s41598-022-14297-5

**Published:** 2022-06-30

**Authors:** Ayano Kabashima, Yuki Matsuo, Saki Ito, Yoshimitsu Akiyama, Takeshi Ishii, Shu Shimada, Atsushi Masamune, Minoru Tanabe, Shinji Tanaka

**Affiliations:** 1 Department of Molecular Oncology, Graduate School of Medicine, Tokyo Medical and Dental University (TMDU), 1-5-45 Yushima, Bunkyo-ku, Tokyo, 113-8519 Japan; 2 Department of Cardiovascular Medicine, Mayo Clinic, Rochester, MN USA; 3 Department of Hepato-Biliary-Pancreatic Surgery, Graduate School of Medicine, Tokyo Medical and Dental University (TMDU), Tokyo, Japan; 4 Division of Gastroenterology, Tohoku University Graduate School of Medicine, Sendai, Japan

**Keywords:** Cancer, Cancer microenvironment, Gastrointestinal cancer, Tumour immunology

## Abstract

Immune checkpoint blockade (ICB) treatment improves the prognosis of several types of solid tumors, however, responsiveness to ICB therapy remains low in pancreatic ductal adenocarcinoma (PDACs), which has a rich tumor microenvironment (TME). The TME is composed of various stromal cells, including cancer-associated fibroblasts (CAFs), which contribute to the establishment of an immunosuppressive microenvironment. The cyclic GMP-AMP synthase (cGAS)-stimulator of interferon genes (STING) pathway is an innate immune pathway that results in the upregulation of immune cell recruiting-cytokines and anti-tumor efficacy. In this study, we aimed to investigate the impact of cGAS-STING expression and the presence of CAFs upon immune cell infiltration in PDACs. cGAS and STING co-expressing PDAC cases showed favorable survival, with many cytotoxic CD8 + T cell infiltrations from the stromal component adjacent to the cancer cells toward cancer cells, but not in cGAS-STING signaling defected PDAC cases. The signatures of tumor-restrain CAFs were expressed in tumors with cGAS-STING signaling. Finally, transwell co-culture experiments demonstrated that immune cell infiltration was impeded by the presence of CAFs, but not by activation of cGAS-STING signaling. In conclusion, pro-infiltration signals, such as cGAS-STING, and characterization of CAFs are crucial in defeating CAF barricades and encouraging immune cell infiltration in PDACs.

## Introduction

Pancreatic ductal adenocarcinoma (PDAC) is the most lethal neoplasm with limited therapeutic options and dismal 5-year survival rate of less than 10%^1,2^. Owing to aggressive progression, only 20–30% of PDAC patients are amenable to resection at the time of diagnosis, despite surgical resection of the primary tumor being the only curative treatment^2^. Although the survival outcomes of PDAC patients have improved with multimodal treatment, the efficacy of systemic therapies remains limited, and the development of highly effective therapies is an urgent issue for PDAC patients.

Immune checkpoint blockade (ICB) has revolutionized cancer therapies and resulted in improved survival outcomes in patients with a wide range of cancers^3^. Nevertheless, the response to ICB in PDACs has been disappointing, partly due to the presence of dense stroma as a tumor-suppressive microenvironment that impedes tumor angiogenesis and therapeutic drug delivery^4–6^.

The cyclic GMP-AMP synthase (cGAS)-STING pathway mediates innate immunity by inducing the production of type -I interferons (IFNs) and various inflammatory cytokines in response to cytoplasmic DNA^7^. An adaptor protein, STING, is activated by cyclic dinucleotides (CDNs), such as 2′,3′-cyclic GMP-AMP synthase (cGAMP), which is generated from cytoplasmic DNA by cGAS, and subsequently activates TANK-binding kinase 1 (TBK1) and interferon regulatory transcription factor 3 (IRF3). The translocation of IRF3 to the nucleus upregulates immune stimulated genes (ISGs) and immune cell-recruiting cytokines, followed by activation and migration of immune cells, including dendritic cells (DCs), T cells, and natural killer (NK) cells^8^. The cGAS-STING pathway is now also recognized as a crucial mechanism that regulates immunity in various tumors^9,10^; however, expression of cGAS and/or STING is frequently lost or downregulated, which causes suppression of the intrinsic cytosolic DNA-mediated innate immune system. Stimulation of this pathway provides the ability to overcome the immunosuppressive environment of tumors and sensitize patients to ICB therapy.

PDAC is also well known for the development of dense fibrosis termed desmoplasia, which is composed of pancreatic stellate cells, fibroblasts, immune cells, and extracellular matrix proteins^11^. In particular, cancer-associated fibroblasts have been considered to not only impede the penetration of drugs and infiltration of anti-tumor immunocompetent cells but also recruit immunosuppressive cells into the tumor microenvironment (TME), resulting in a contribution to disease progression and therapeutic resistance by maintaining the TME immunosuppressive^12–15^. Recent studies have demonstrated that cancer-associated fibroblasts (CAFs) consist of various functionally heterogeneous subsets that either promote or restrain tumor progression and are termed tumor-promoting CAFs (pCAFs) and tumor-restrain CAFs (rCAFs), respectively. In this study, we explored the relevance of CAFs and cGAS-STING signaling in facilitating immune cell infiltration in PDACs. Our results demonstrated that cGAS-STING-expressing tumors displayed elevated markers of tumor-suppressive CAFs, as well as strong infiltration of anti-tumor immune cells in the stromal component of the tissues. Moreover, transwell co-culturing experiments demonstrated that the presence of CAFs attenuated immune cell infiltration toward cancer cells, but was abolished by activation of cGAS-STING signaling. These results suggest that the complementation of cGAS-STING signaling in the tumor would be a promising therapeutic strategy for defeating CAF barricades in PDACs.

## Results

### Downregulation of cGAS expression correlates with poor prognosis in PDACs

**Figure 1:**
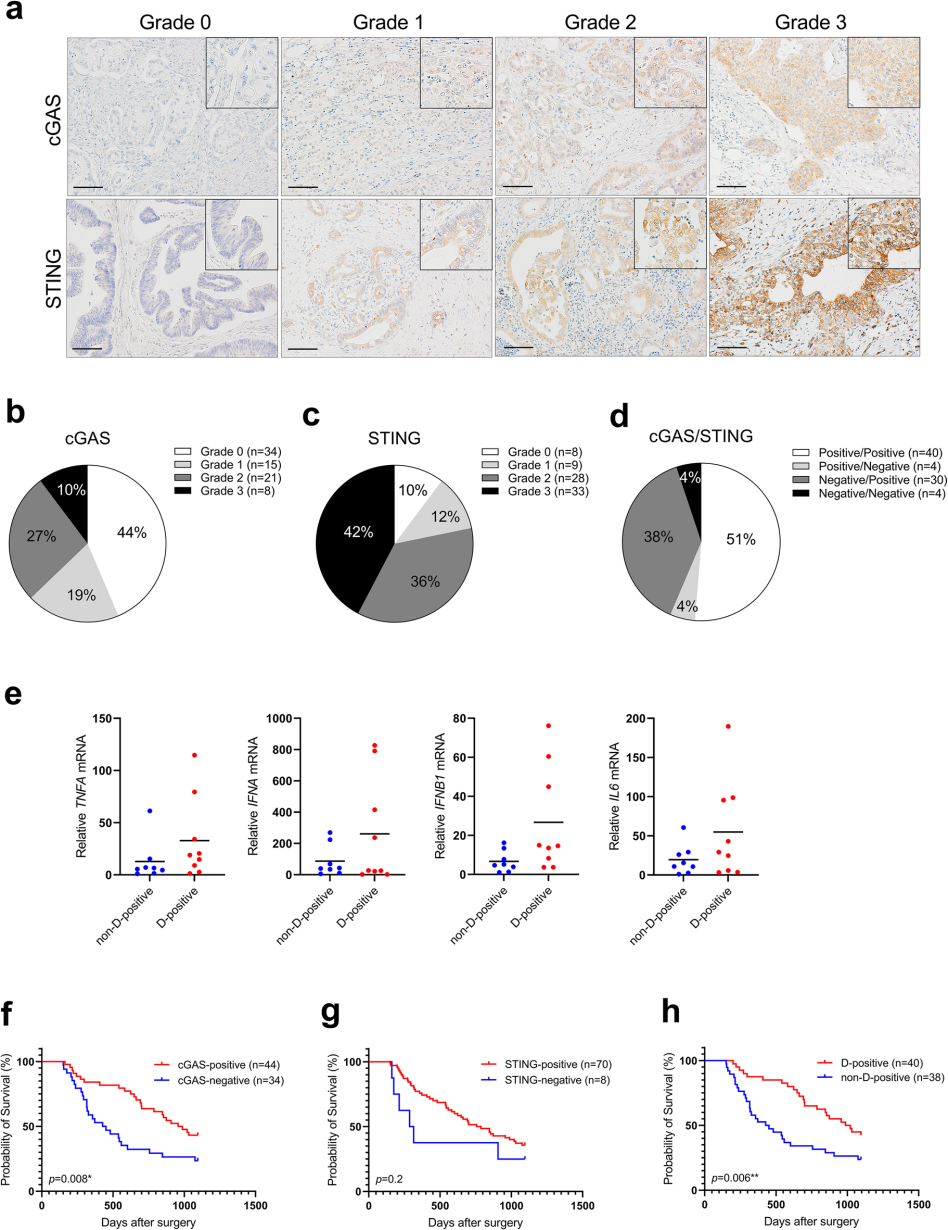
Downregulation of cGAS-STING expression correlates with poor prognosis in PDACs. (**a**) The representative immunohistochemical staining of cGAS and STING in each expression grade. (**b**) Percentage of each cGAS expression grade in 78 PDAC cases. (**c**) Percentage of each STING expression grade in 78 PDAC cases. (**d**) Percentage of cGAS/STING expression in each case. cGAS and STING double positive as Positive/Positive; only cGAS positive as Positive/Negative; only STING positive as Negative/Positive; and both negative as Negative/Negative. (**e**) qPCR analysis for target genes of cGAS-STING pathway in cGAS-STING double-positive (D-positive) cases vs other cases (non-D-positive); including cGAS or STING single-positive, or double-negative. (**f**) Kaplan–Meier analysis in cGAS-positive (Grade 1, 2, 3) cases vs cGAS-negative (Grade 0) cases. (**g**) Kaplan–Meier analysis in STING-positive (Grade 1, 2, 3) cases vs STING-negative (Grade 0) cases. (**h**) Kaplan–Meier analysis in D-positive cases vs non-D-positive cases. Scale bar: 100 µm. **p < 0.01, *p < 0.1. Statistical significance in the Kaplan–Meier analysis was determined using the log-rank test. This figure was created using GraphPad Prism 9.3.0 (https://www.graphpad.com/scientific-software/prism/).

The importance of cGAS-STING signaling in cancer has been evidenced in many preclinical models, both in vivo and in vitro^16–19^. STING expression is often lost or downregulated in gastrointestinal tumors^17–19^. To assess the expression levels of cGAS and STING in PDACs, we first performed an immunohistochemical analysis of 78 PDAC clinical specimens from surgical resections at Tokyo Medical and Dental University (TMDU) Hospital. Representative staining of cGAS and STING for each expression grade is shown in Fig. 1a. cGAS was predominantly localized in both the cytoplasm and peri-nucleus; STING was predominantly localized in the cytoplasm. The expression of cGAS was found only in cancer cells, whereas STING was found predominantly in cancer cells. However, in several cases, it was found predominantly in the surrounding stromal tissue (Supplemental Fig. 1a). Double immunostaining for SOX9 to recognize cancer cells and for STING revealed that there are two types of STING-positive cells in the stromal compartment: SOX9 positive/STING positive cells thought to be undergoing epithelial-to-mesenchymal transition (EMT) and SOX9 negative/STING positive cells (Supplemental Fig. 1b), and those with STING expression were considered STING-positive regardless of the expression type in this study. Specimens that were found to be expressed in 10% or more of the entire microscopic field were graded from Grade 1 to Grade 3 according to the expression levels, and specimens with less than 10% positive areas were graded Grade 0. Of all patients, cGAS- and STING-positive cases were 56% and 90%, respectively (Fig. 1b,c). Unlike most gastrointestinal tumors, STING expression was predominantly preserved in most cases. When observed at the same location in the tissue, 51% of the cases were positive for both cGAS and STING (positive/positive), 4% were positive for only cGAS (positive/negative), 38% were positive for only STING (negative/positive), and 4% were negative for both (negative/negative; Fig. 1d). cGAS and STING function together to activate this pathway. We divided the cases into two groups: cGAS and STING double-positive (hereafter referred to as D-positive) and others (including cGAS or STING single-positive and double-negative, hereafter referred to as non-D-positive). The target genes of the cGAS-STING pathway were evaluated by quantitative PCR in 17 PDAC formalin-fixed paraffin-embedded (FFPE) samples. The characteristics of the 17 FFPE samples are listed in Table 1. Most D-positive cases had a higher expression of all target genes than that in the non-D-positive cases, demonstrating that cGAS-STING signaling was more activated in D-positive cases (Fig. 1e).

**Table 1:** Biological features of 17 FFPE samples.

Patient#	Age	Sex	cGAS Grade	STING Grade	D-positive?	Survival (days)	F-Grade
1	68	M	2	2	Y	2078	3
2	64	F	0	3	N	327	2
3	80	M	0	0	N	286	2
4	67	F	2	3	Y	1275	2
5	47	M	2	2	Y	1109	2
6	71	M	0	1	N	175	1
7	71	M	2	2	Y	698	2
8	72	F	0	3	N	150	2
9	70	F	1	3	Y	1524	3
10	67	F	0	1	N	154	3
11	61	F	1	2	Y	1031	3
12	68	M	0	3	N	213	3
13	78	F	2	3	Y	1899	2
14	70	M	0	0	N	2307	2
15	75	M	0	3	N	544	3
16	57	M	2	3	Y	1967	2
17	86	F	2	2	Y	1941	1

*M,* Male; *F,* Female; *D-positive,* cGAS and STING expression double-positive; *F-Grade,* fibrosis grade in the tissue.

Next, we evaluated the correlation between cGAS/STING expression and clinicopathological factors, including overall survival (OS) in all patients with PDAC. Kaplan–Meier analysis demonstrated that there was no significant difference in overall survival between STING-negative and STING-positive patients; however, cGAS-negative patients showed significantly lower survival outcomes than that by the cGAS-positive patients (Fig. 1f,g). Furthermore, the survival rate of D-positive patients was higher than that of non-D-positive patients (Fig. 1h). As indicated in Table 2, univariate Cox proportional models showed that tumor size (HR 1.95; 95% CI 1.09–3.48; p = 0.03), N-status (HR 2.29; 95% CI 1.17–4.48; p = 0.009), Stage (HR 2.45; 95% CI 1.19–5.04; p = 0.008), and cGAS-STING expression level (HR 0.47; 95% CI 0.27–0.82; p = 0.008) were associated with overall survival in PDAC patients. A further estimate by multivariable Cox regression analysis demonstrated that co-expression of cGAS and STING was associated with survival outcomes in PDAC patients after adjustment for age and stage (HR 1.93; 95% CI 1.09–3.42; p = 0.02).

**Table 2:** Clinicopathological characteristics associated with overall survival outcomes in PDAC patients-Univariate Cox proportional hazard models.

Factors	Cut-off	Univariate analysis		
		HR	95% CI	*p*-value
Age, years	> 70	1.06	0.61–1.84	0.80
Sex	Male	0.76	0.43–1.32	0.30
Tumor size, mm	> 40	1.95	1.09–3.48	0.03*
CEA, ng/ml	> 5	1.22	0.67–2.20	0.50
CA19-9, U/ml	> 80	1.01	0.14–7.43	0.50
Differentiation, poor**	Present	1.71	0.98–3.00	0.06
Venous invasion	Present	1.39	0.34–5.71	0.60
Lymphatic vessel invasion	Present	1.37	0.77–2.41	0.30
UICC T-status	≥ T3	1.37	0.77–2.41	0.30
UICC N-status	> N1	2.29	1.17–4.48	0.009*
UICC M-status	M1	1.43	0.61–3.37	0.40
Stage	≥ III	2.45	1.19–5.04	0.008*
cGAS-STING expression	Positive	0.47	0.27–0.82	0.008*

*UICC,* Union for International Cancer Control; *HR,* Hazard Ratio; *95% CI,* 95% Confidence Interval; *Indicates statistical significance, < 0.05; **Indicates the cases which contain poorly differentiated component in the specimen.

### cGAS and STING double expression is associated with the infiltration of anti-tumor immunocompetent cells in PDAC tissues

**Figure 2:**
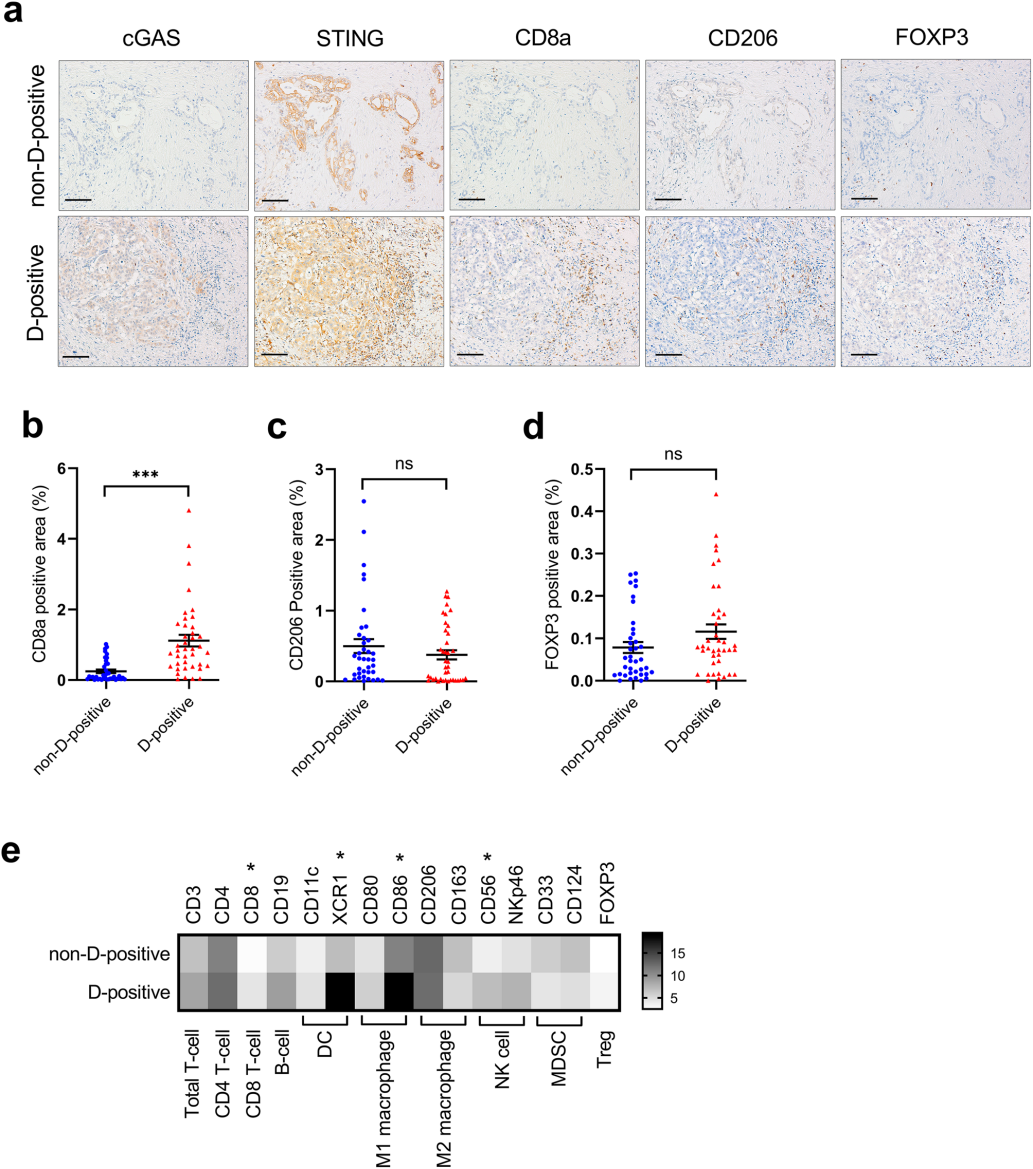
The infiltration of anti-tumor immune cells is enhanced in the cGAS-STING double-expressing PDAC tumors. (**a**) Immunohistochemical staining for cGAS, STING, CD8a, CD206, and FOXP3 in representative non-D-positive and D-positive cases. (**b**) The percentage of CD8a expression positive area in cGAS-STING double-positive (D-positive) tissues versus other (non-D-positive) PDAC tumors. (**c**) The percentage of CD206 expression positive area in cGAS-STING double-positive (D-positive) tissues versus other (non-D-positive) PDAC tumors. (**d**) The percentage of FOXP3 expression positive area in cGAS-STING double-positive (D-positive) tissues versus other (non-D-positive) PDAC tumors. (**e**) RT-qPCR analysis of immune cell markers in 17 FFPE PDAC tissues. Heat map showing the fold increase (versus the lowest value of each gene). Scale bar: 100 µm. Each positive area in the immunohistochemical staining was assessed by morphometry from 3 random microscopic fields (10 × objective). ***p < 0.001. *ns* non-significant. Statistical significance was determined using the 2-tailed *t* test. This figure was created using GraphPad Prism 9.3.0 (https://www.graphpad.com/scientific-software/prism/).

Activated cGAS-STING signaling transcriptionally provokes the expression of various cytokines and subsequent recruitment of immune cells to cancer cells^20^. Given that the presence of cGAS-STING signaling was strongly correlated with survival in patients with PDAC, we next sought to evaluate whether there is a relationship between the infiltration of tumor-associated immune cells and the presence of cGAS-STING signaling. In the non-D-positive cases without cGAS expression as shown in the upper panels of Fig. 2a, only a small number of cytotoxic CD8 + T cells were observed, while in the D-positive cases expressing both cGAS and STING, a large number of cytotoxic CD8 + T cells infiltrated the vicinity and inside of the tumor in many cases compared to non-D-positive cases (Fig. 2a, lower panels). Accordingly, quantitative evaluation of all 78 cases demonstrated that the infiltration of cytotoxic CD8 + T cells in D-positive cases was approximately fourfold higher than that in non-D-positive cases (Fig. 2b). In the TME of highly lethal malignancies, such as PDACs, immune system suppression by infiltrated immunosuppressive cells including tumor-associated macrophages (TAMs), regulatory T cells (Tregs), and myeloid-derived suppressor cells (MDSCs), is often involved in tumor progression^12,13^. To explore whether the presence of cGAS-STING signaling correlates with the infiltration of immunosuppressive cells, CD206 (for M2 macrophages as TAMs), and FOXP3 (for Tregs) expression in clinical specimens were evaluated by immunohistochemical analysis. Both were expressed in the vicinity of the cancer cells and the stromal region, though FOXP3 positive cells were observed in very small numbers when compared to CD206 positive cells (Fig. 2a). The amount of infiltration of these immunosuppressive cells was variable among cases, although there was no significant difference between D-positive and non-D-positive cases in the quantitative evaluation. The percentage of positive area demonstrated that the tendency of CD206 was less in D-positive cases than that in non-D-positive cases, and FOXP3 was less in non-D-positive than in D-positive cases (Fig. 2a,c,d). We also explored tumor-associated immunocompetent cell infiltration levels in clinical specimens by evaluating the mRNA levels of 17 PDAC FFPE samples. Each fold change was determined by normalization with the minimum Ct value of each group. Consistent with the results of immunohistochemical analysis, cytotoxic CD8 + T cells were higher in D-positive samples than in non-D-positive samples, and anti-tumor immunocompetent cells including dendritic cells (DCs), and NK cells were also higher in D-positive samples than in non-D-positive samples (Fig. 2e). Meanwhile, myeloid-derived suppressor cells (MDSCs) were minor in D-positive samples, suggesting a strong association between the presence of cGAS-STING signaling and the tumor immune microenvironment (TIME).

### Knock-out of cGAS attenuates cytokine expressions as well as immune cell migrations toward cancer cells

**Figure 3:**
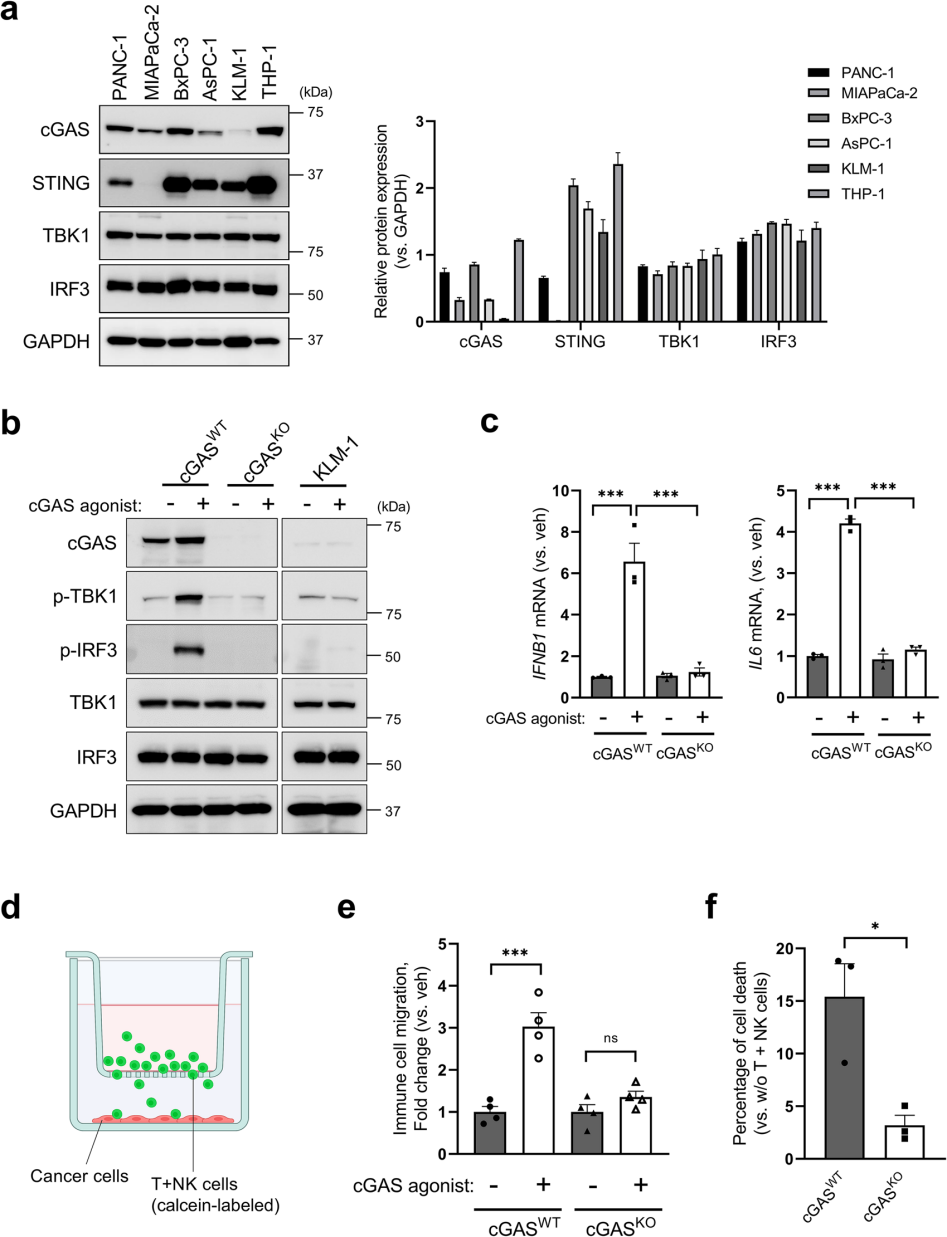
Knock-out of cGAS attenuates cytokine expressions and subsequent immune cell migrations toward cancer cells. (**a**) Immunoblot (left panel) and densitometric (right panel) analysis of cGAS-STING signaling associated proteins. THP-1 human acute monocytic leukemia cells were employed as a positive control for cGAS-STING signaling. Densitometric data for each protein expression was normalized to GAPDH. (**b**) cGAS-STING signaling activation by cGAS agonist, G3-YSD. cGAS^WT^ BxPC-3, cGAS^KO^ BxPC-3, and THP-1 cells were treated with or without G3-YSD (1 µg/mL) for 5 h by lipofection. (**c**) IFNB1 and IL6 mRNA levels were assessed by RT-qPCR. Cells were treated with or without G3-YSD (1 µg/mL) for 24 h. (**d**) A scheme of transwell chamber for an immuno-cancer cells coculture, depicted using BioRender. (**e**) Immune cell migration assay in BxPC-3 cells with or without cGAS agonist. (**f**) Cell death rate of cGAS^WT^ BxPC-3 and cGAS^KO^ BxPC-3 cells caused by immune cell coculturing. The bar graphs represent mean ± standard error of the mean (SEM) for at least three independent experiments. *p < 0.1, ***p < 0.001. *ns* non-significant. Statistical significance was determined using the 2-tailed *t* test or one-way analysis of variance (ANOVA) followed by Tukey’s test. This figure was created using GraphPad Prism 9.3.0 (https://www.graphpad.com/scientific-software/prism/).

Next, we examined cGAS/STING expression in the PDAC cell lines. As shown in Fig. 3a, immunoblot analysis demonstrated that cGAS expression was less in several cell lines (for MIAPaCa-2, AsPC-1, and KLM-1) compared to the expression level in THP-1 cells, as a positive control. The introduction of the cGAS agonist G3-YSD increased the phosphorylation of TBK1 and IRF3, which is a transcription factor that induces type-I interferon and other immune cell-recruiting cytokines in cGAS^WT^ BxPC-3 cells (Fig. 3b). Deletion of gene encoding *cGAS* using CRISPR/Cas9 diminished the phosphorylation of TBK1 and IRF3 in BxPC-3 cells. Likewise, these signaling factors were not sufficiently phosphorylated in KLM-1 cells with low cGAS expression (Fig. 3b). Consistent with the phosphorylation levels of signaling factors in immunoblot analysis, IRF3 targeting cytokines, *IFNB1* and *IL6* mRNA expression levels were upregulated in response to the introduction of the cGAS agonist in cGAS^WT^ BxPC-3 cells, but not in cGAS^KO^ BxPC-3 cells (Fig. 3c). We also sought to evaluate the immunocompetent cell-attractive capacity of the cancer cells based on previous reports^21,22^. Calcein-AM-labeled T + NK cells plated in 3.0 µm pored-transwell chambers migrated towards cancer cells in the lower chamber, as shown in Fig. 3d. Counting of calcein-AM-positive immune cells in the lower chamber demonstrated that signal activation by introducing cGAS agonist attracted 3.0-fold higher immune cells compared to vehicle in cGAS^WT^ BxPC-3 cells, but only a 1.4-fold increase in cGAS^KO^ BxPC-3 cells (Fig. 3e). Furthermore, an immune cell-killing assay was performed using cGAS^WT^ and cGAS^KO^ BxPC-3 cells. To vaccinate DCs, cell lysates were prepared from each cell by repeating freeze–thaw cycles and sonication and treated with matured DCs differentiated from monocytes in PBMCs. T + NK cells primed with vaccinated DCs migrated toward the lower chamber and killed cancer cells. Corresponding to the number of migrated immune cells, the cell viability assay revealed that approximately fivefold more cancer cells were dead in cGAS^WT^ cells compared to cGAS^KO^ cells (Fig. 3f). Considered together, the presence of cGAS is a critical factor for the augmentation of immune cell recruitment in PDACs, which frequently lose or downregulate cGAS expression.

### The characteristics of tumor-suppressive CAFs were preserved in PDAC tissues with the presence of cGAS-STING signaling

**Figure 4:**
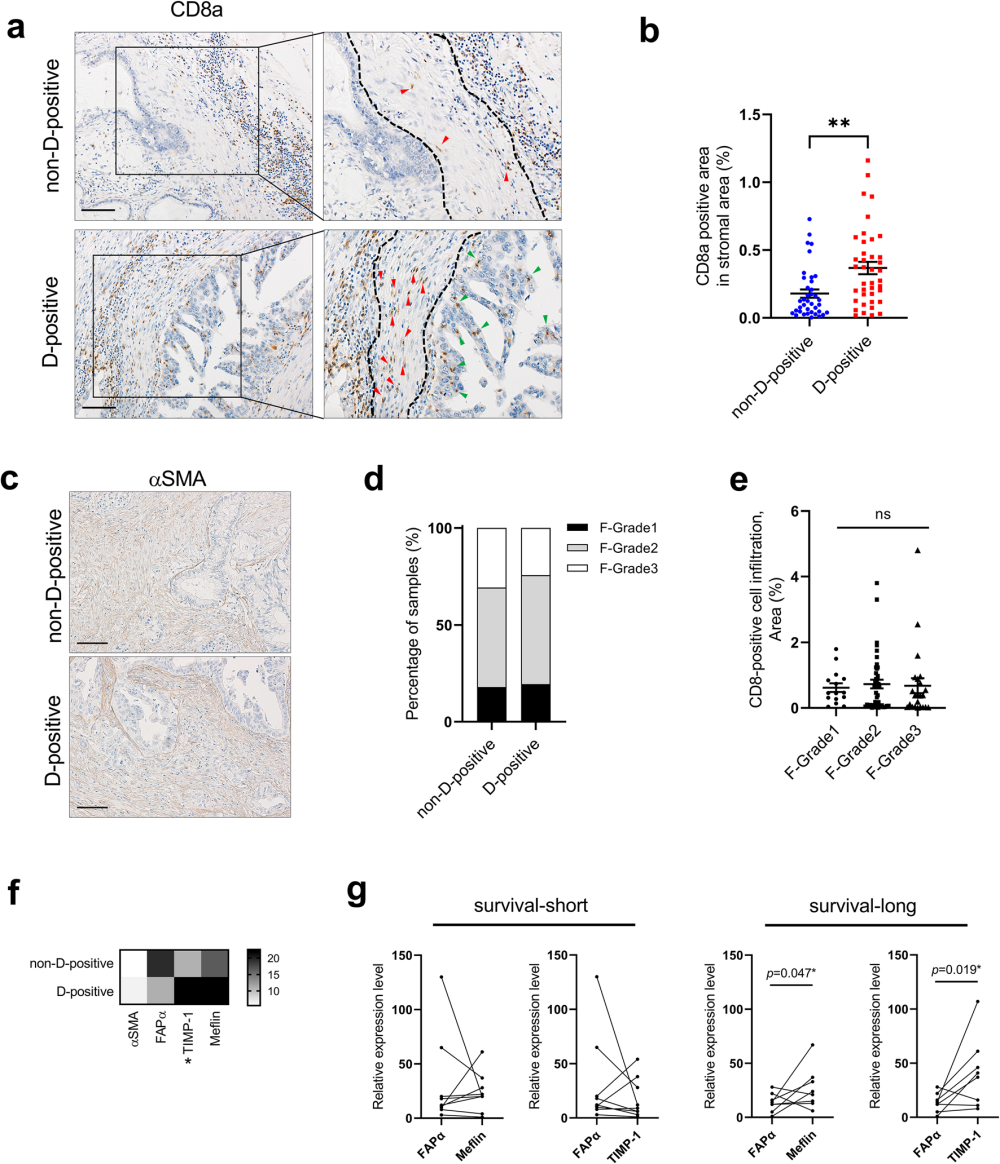
The characteristics of tumor-suppressive CAFs were preserved in PDAC tissues with presence of cGAS-STING signaling. (**a**) Infiltration of cytotoxic CD8 + T cells cells in representative non-D-positive and D-positive tissues. There are fewer cytotoxic CD8 + T cells in the stromal compartment adjacent to cancer cells in non-D-positive tissue (red arrows in upper panel). By contrast, in D-positive cases, a large number of cytotoxic CD8 + T cells continuously infiltrate from the stromal compartment to the cancer cells (red and green arrows in lower panel). (**b**) Quantification of CD8a-positive cells infiltrating within the stromal component adjacent to cancer cells in non-D-positive and D-positive cases. (**c**) The representative immunohistochemical staining of alpha-smooth muscle actin in non-D-positive cases (upper) and D-positive cases (lower). (**d**) The percentage of samples among each fibrosis grade in non-D-positive cases and D-positive cases. (**e**) The comparison of CD8a staining positive area among each fibrosis grade. (**f**) RT-qPCR analysis of CAF markers in 17 FFPE PDAC tissues. Heat map showing the fold increase (versus the lowest value of each gene). (**g**) Correlation of overall survivals (OS short, < 1095 days; OS long, > 1095 days) and expression levels of FAPa vs Meflin or TIMP-1 in 17FFPE PDAC samples. Scale bar: 100 µm. Each positive area in the immunohistochemical staining was assessed by morphometry from 3 random microscopic fields (10 × objective). The bar graph represents mean ± standard error of the mean (SEM) for at least three independent experiments. Statistical significance was determined using the one-way analysis of variance (ANOVA) followed by Tukey’s test. **p < 0.01. *ns* non-significant. This figure was created using GraphPad Prism 9.3.0 (https://www.graphpad.com/scientific-software/prism/).

Lethal malignancies often harbor abundant tumor-specific stromal cells, which decrease the therapeutic effects and act as a barrier to the accessibility of anti-tumor effectors, including drugs and immune cells^23,24^. Although the infiltration of cytotoxic CD8 + T cells into the stromal component was observed in both non-D-positive and D-positive cases (Fig. 4a, red arrows), in the non-D-positive cases, large numbers of cytotoxic CD8 + T cells were clustered together in a location away from the cancer cells and small numbers were present in the stroma adjacent to the cancer cells (Fig. 4a upper panel), whereas, in the D-positive cases, most cytotoxic CD8 + T cells continuously infiltrated from the adjacent stroma to the inside of the cancer tissue (Fig. 4a, green arrows in lower panel). Quantitative analysis of CD8 + T cells in the stromal component adjacent to the cancer cells (surrounded by dashed lines) revealed that a higher number of T cells significantly infiltrated in the stroma for D-positive cases (Fig. 4b). To investigate the factors determining the susceptibility to infiltrate into the stromal component, we first examined the correlation between the amount of CAFs and the presence or absence of cGAS-STING signaling. Alpha smooth muscle actin (αSMA) has been studied as one of the standard marker for identifying CAFs ^24^. We first performed an immunohistochemical analysis of αSMA and determined the histological grade based on the amount of positive cells in the 78 PDAC samples. As the representative staining of αSMA is shown in Fig. 4c, no staining difference was observed between D-positive and non-D-positive cases, also, the percentage of each histological grade was approximately the same between the groups (Fig. 4d). Furthermore, the comparison of the cytotoxic CD8 + T cell infiltration ratio also showed no significant difference among the fibrosis grades (Fig. 4e). Having demonstrated that neither the presence of cGAS-STING signaling nor the infiltration of cytotoxic CD8 + T cells depends on the fibrosis grade of the tissues, we next investigated the characteristics of CAFs in PDAC tumors. Recently, a growing body of evidence has shown that CAFs are composed of a variety of subsets with functional heterogeneity: tumor-promoting CAFs (pCAFs), tumor-restrain CAFs (rCAFs), and neutral CAFs (nCAFs)^24^. Those specific markers are still being investigated, but in particular, rCAFs have been shown to express TIMP-family proteins and Meflin, which maintain the undifferentiation feature of CAFs, loss of those expressions results in the acquisition of activated CAFs phenotypes which facilitate tumor progression including tumor proliferation, metastasis, and therapeutic resistance^25,26^. Quantitative PCR in 17 FFPE PDAC samples showed that the expression of both TIMP-1 and Meflin was higher in D-positive cases than in non-D-positive cases; while FAPα expression tended to be higher in non-D-positive cases (Fig. 4f). Furthermore, the expression comparison of FAPα versus Meflin or TIMP-1 in the same tumor of the 17 FFPE PDAC samples showed that higher-Meflin and/or higher-TIMP-1 than FAPα is involved in prolonged survival (Fig. 4g). These results suggest that cGAS-STING signaling activity in PDAC may correlate with the properties of CAFs.

### Loss of cGAS-STING signaling attenuated T + NK cell infiltration toward cancer cells and subsequent cytotoxicity

**Figure 5:**
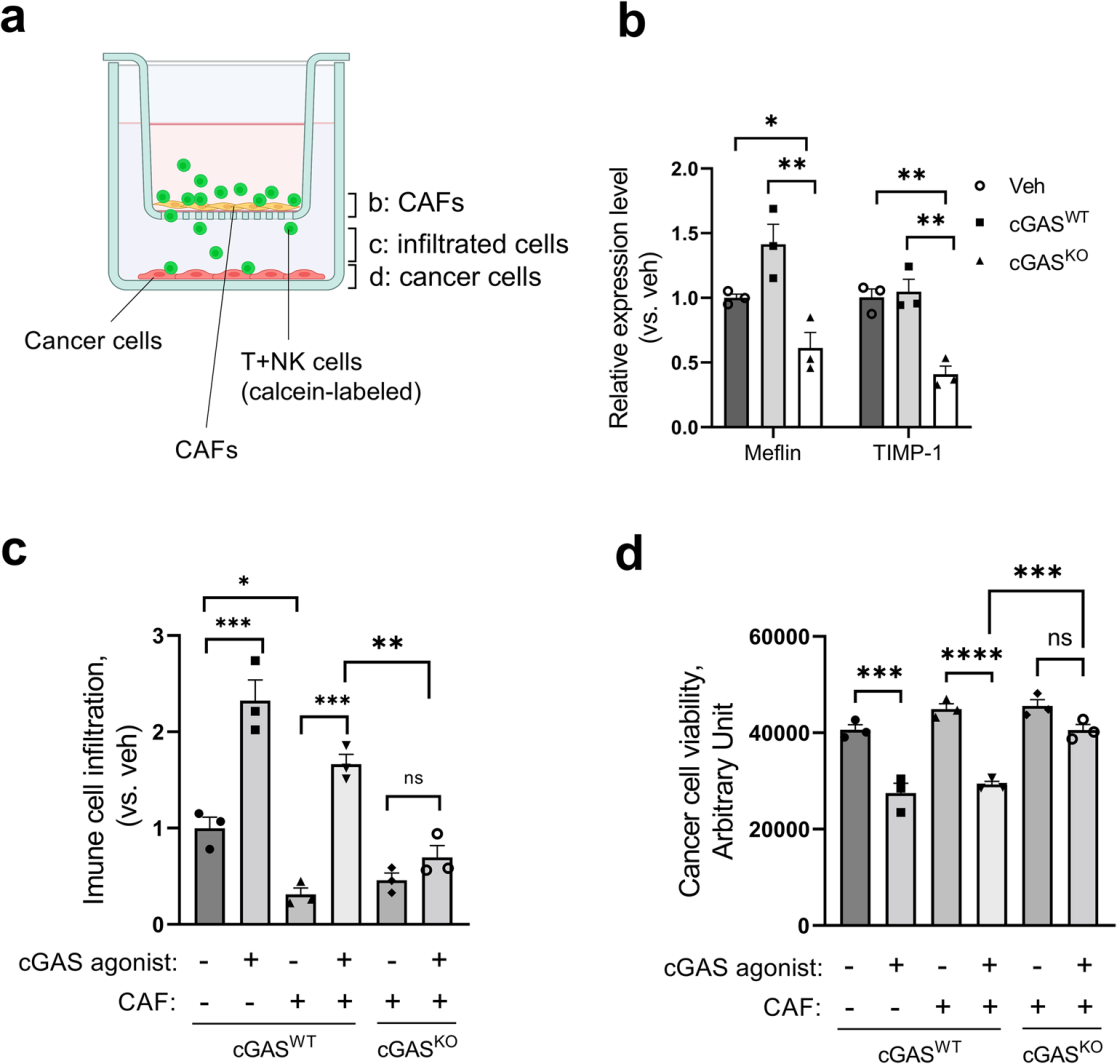
Loss of cGAS-STING signaling attenuated T + NK cell infiltration toward cancer cells and subsequent cytotoxicity. (**a**) A scheme of transwell chamber for fibroblast-immune-cancer cells co-culture, depicted using BioRender. The labeled compartments on the chamber scheme are corresponded to the analyzed samples of each following figure. (**b**) Expression of Meflin and TIMP-1 in CAFs following co-culture with cGAS^WT^ BxPC-3 or cGAS^KO^ BxPC-3. (**c**) Immune cell infiltration assay in co-culture system of cGAS^WT^ or cGAS^KO^ BxPC-3 cells with CAFs and T + NK cells. (**d**) Cell viability assay in co-culture system of cGAS^WT^ or cGAS^KO^ BxPC-3 cells with CAFs and T + NK cells. The bar graph represents mean ± standard error of the mean (SEM) for at least three independent experiments. Statistical significance was determined using the one-way analysis of variance (ANOVA) followed by Tukey’s test. *p < 0.1, **p < 0.01, ***p < 0.001, ****p < 0.0001. *ns* non-significant. This figure was created using GraphPad Prism 9.3.0 (https://www.graphpad.com/scientific-software/prism/).

We examined whether the presence or absence of cGAS-STING signaling could alter the characteristics of CAFs. Following stimulation of cGAS^WT^ BxPC-3 or cGAS^KO^ BxPC-3 cells with a cGAS agonist, they were co-cultured with CAFs and T + NK cells pre-treated with conditioned medium of the corresponding cancer cells, as shown in Fig. 5a. After culturing, CAF mRNA in the upper chamber was collected and CAF properties were evaluated by qPCR. As shown in Fig. 5b, co-culture with cGAS^WT^ BxPC-3 slightly upregulated or maintained the expression of both Meflin and TIMP-1, while cGAS^KO^ BxPC-3 reduced the expression of both to 41% and 39% of cGAS^WT^, respectively. Whereas FAPα did not show significant expression changes in co-culture with cGAS^WT^ BxPC-3 or cGAS^KO^ BxPC-3 cells. These results suggested that the presence of cGAS-STING signaling in cancer cells responsible for the maintenance of tumor-suppressive properties in the surrounding CAFs.

In further experiments, we assessed the immunocompetent cell infiltration ability mediated by cGAS-STING signaling in the absence or presence of CAFs. The presence of CAFs resulted in a 70% reduction of immune cell infiltration compared to the absence of CAFs; however, only a 28% reduction was observed in cGAS agonist-treated cGAS^WT^ groups with CAFs. Additionally, cGAS-STING-mediated infiltration was abolished in cGAS^KO^ BxPC-3cells (Fig. 5c). Furthermore, we evaluated the viability of the cancer cells in this co-culture system. Consistent with the results in Fig. 5c, cGAS agonist stimulation in cGAS^WT^ BxPC-3 cells induced cell death associated with immune cell infiltration even in the presence of CAFs, with a 35% decrease in viability compared with no agonist stimulation. In contrast, immune cell infiltration and cell death were minimal in cGAS^KO^ BxPC-3 cells (Fig. 5d).

These findings suggest that not all CAFs reduce immune cell infiltration, and that the presence or absence of cGAS-STING signaling in PDAC, and the resulting CAF properties may impact immune cell infiltration against cancer cells.

## Discussion

The principal findings of this study provide new insights into the relevance of cGAS-STING immuno-modulatory signaling and CAFs in PDAC. Our results indicate the following: (i) STING expression is observed in 90% of PDAC cases, and the overall survival of PDAC patients is higher in cGAS and STING double-expressing tumors; (ii) in the tissues with cGAS-STING double-positive, there is a continuous infiltration of cytotoxic CD8 + cells from stromal to cancerous tissues, and they contain more rCAFs; and (iii) in the co-culture system, cGAS-STING signaling facilitates immune cell infiltration and subsequent cancer cell death by maintaining rCAF properties of CAFs. These results are further discussed below.

Recent research has revealed the importance of pattern recognition receptor (PRR) signaling in cancer, such as cGAS-STING, which is triggered by cytoplasmic DNA and promotes the secretion of cytokines involved in immune cell recruitment, and complementing this signaling is anticipated to strengthen the anti-tumor effect^9,20,27^. Nevertheless, the expression of the signaling components cGAS and STING is often decreased in tumors, especially in gastrointestinal cancers, where STING expression is often downregulated. In this study, we observed that STING expression was preserved in 90% of PDAC cases, whereas, cGAS expression was lost in 44% of the cases. Previously, Xia et al. demonstrated that cGAS expression was undetectable in 31% of colorectal cancer tissues, with no mutations or deletions, instead hypermethylation. Indeed, cGAS expression was recovered with the demethylating agent 5-aza-2’-deoxycytidine (5AZADC)^28^. There was no significant correlation between STING expression and overall survival in this study, possibly due to the small number of STING-negative PDAC patients, but there was a tendency for correlation. In fact, the prognostic correlations of cGAS-STING double-positive cases were greater than those of cGAS or STING expression alone (Fig. 1f–h).

In some cases, STING expression was not limited in cancer cells but was also found in stromal tissues. This included cells that underwent EMT and acquired mesenchymal properties; however, in several cases, stromal cells were predominantly expressed. Arwert et al. reported that transcytosis of the cytoplasm from cancer cells into fibroblasts resulted in STING activation and subsequent cytokine expression^29^. Furthermore, several other studies have shown that STING stimulators are provided by neighboring cells^30,31^. Interactions between cancer cells and fibroblasts may also be an attractive tool for modulating tumor immunology. In our study, cytotoxic CD8 + T cells were distributed within the stromal tissue in stromal-STING-dominant cases, and their recruitment to cancer cells was lower than in cases which cGAS-STING was expressed in cancer cells. However, there were only six obvious stromal-STING-dominant cases in this experiment, which is not enough evidence, and further investigation of the effects of these cases on immune cell recruitment would be fascinating in combination with transcytosis.

Pancreatic cancer tissues are rich in stromal cells and are representative of desmoplastic malignancies. Various types of cells comprise the tumor stroma and construct a unique TME in PDACs, the major component of which, CAFs, have been shown in recent reports to include heterogeneous subsets. In many desmoplastic tumors, CAFs are thought to construct an immunosuppressive milieu by remodeling the ECM and recruiting immunosuppressive cells, which impedes the recruitment of anti-tumor immune cells^23,24^. In the present study, we observed that in tumor tissues without cGAS-STING signaling, cytotoxic CD8 + T cells are more distant from the tumor periphery, and their infiltration within the adjacent stromal tissue and toward the cancer cells is suppressed (Fig. 4a,b). Although the infiltration level of cytotoxic CD8 + T cells into the tumor tissue did not differ among the fibrosis grades, the marker of rCAF, which functions as tumor suppressive, was highly expressed in the presence of cGAS-STING signaling tumors. In vitro co-culture experiments utilizing transwell chambers demonstrated that the absence of cGAS-STING signaling downregulates Meflin and TIMP-1, which are both responsible for rCAF function, and that the inhibition of immune cell infiltration toward cancer cells by the presence of CAF was diminished through activation of cGAS-STING signaling. These results are also supported by some of the following reports: Carstens et al. demonstrated that Collagen I and αSMA + fibroblasts did not correlate with less accumulation of T cells, indicating that the presence of stromal cells does not always interfere with T cell infiltration^32^. Another study also reported that deactivation of pancreatic stellate cells (PSCs) reduced CD8 + T cell adhesion and migrated toward PSCs instead, enhanced juxta-tumoral infiltration of CD8 + T cells in PDAC^33^. Our data, as well as those reported by others, imply that CAF heterogeneity may modulate the suppression or promotion of immune cell infiltration. Our results also indicated that certain signals, such as the cGAS-STING signal, are important for immune cells to overcome the stromal barrier and promote their infiltration. However, the co-culture system employed in this study still needs to be improved, as it does not accurately recapitulate the more complex TMEs of actual PDACs, including collagen barriers and immune exhaustion. Investigating how cGAS-STING signaling modulates interactions in the TIME by employing culture systems that mimic the in vivo environment will be an additional challenge.

In addition, the Hedgehog (Hh) signaling ligands are expressed by pancreatic epithelial cells which are known to be involved in PDAC development^34,35^. In PDAC, the release of Hh ligands also functions in a paracrine manner, acting not only on cancer cells but also on the surrounding stromal cells^36^. Hh ligands are crucial for activating fibroblasts into CAFs, but it is currently unknown whether Hh signaling affects the ratio of pCAF to rCAF subpopulations in stroma^4^. Our results suggest that the loss of cGAS-STING signaling attenuates the characteristics of rCAFs. However, it remains unclear whether this is due to a direct effect of cGAS^KO^ cancer cells or an indirect effect of recruited immune cells. Further studies are needed to uncover the mechanism that determines the characteristics of CAFs, including cGAS-STING signaling.

Since the presence of abundant stroma is a potential barrier in PDAC and diminishes the efficacy of ICB, establishing therapeutic strategies for targeting tumor-promoting CAFs is an essential challenge. We demonstrated that the activation of cGAS-STING signaling is important for immune cell infiltration as well as the characteristics of CAFs. Further characterization of heterologous CAFs and complementation of cGAS-STING signaling will encourage immune cells to overcome CAFs, which could ultimately improve the sensitivity of PDACs to ICBs.

## Materials and methods

### Patients and samples

All clinical specimens were obtained from 78 PDAC patients who underwent surgical resection at Tokyo Medical and Dental University (TMDU) Hospital (Tokyo, Japan) in the periods 2010–2020. Clinical data, including age, sex and etiology were obtained from medical records and are shown in Table 3. No cases underwent neoadjuvant therapy, while postsurgical chemotherapy was administered in all cases. The study was performed in accord with the Declaration of Helsinki and was approved by the TMDU ethics committees (Institutional Review Board #G2017-018). All human subjects gave written informed consent for participation in medical research.

**Table 3:** Clinicopathological backgrounds of 78 PDAC samples.

Clinicopathological factors	cGAS-STING signal		*p*-value
	Non-D-positive (n = 38)	D-positive (n = 40)	
Age	67.3 ± 10.0	69.1 ± 8.4	0.38
Sex, male (%)	19 (50)	30 (75)	0.02
Tumor size, mm	37.1 ± 12.8	31.9 ± 17.5	0.14
Serum CEA, ng/ml (range)	3.8 (2.4, 5.0)	3.6 (2.4, 6.6)	0.52
Serum CA19-9, U/ml (range)	135 (26, 457)	87 (36, 297)	0.68
Differentiation, poor**, n (%)	15 (39)	14 (35)	0.68
Venous invasion, n (%)	35 (92)	39 (98)	0.35
Lymphatic invasion, n (%)	23 (61)	22 (55)	0.62
**UICC T-status, n (%)**			0.69
T0	0 (0)	0 (0)	
T1	3 (8)	8 (20)	
T2	21 (57)	21 (50)	
T3	13 (35)	12 (30)	
T4	0 (3)	0 (0)	
**UICC N-status, n (%)**			0.29
N0	10 (26)	15 (37)	
N1	13 (34)	18 (45)	
N2	15 (39)	7 (18)	
UICC M-status, M1, n (%)	4 (10)	3 (8)	0.71
**Stage, n (%)**			0.39
IA	2 (5)	8 (20)	
IB	7 (18)	5 (13)	
IIA	1 (3)	1 (3)	
IIB	10 (26)	16 (40)	
III	11 (29)	7 (18)	
IV	7 (18)	3 (8)	

*UICC,* Union for International Cancer Control; *D-positive,* cGAS and STING expression double-positive; **Indicates the cases which contain poorly differentiated components in the specimen. Continuous variables were summarized as a mean ± SD (standard deviation) or median (interquartile range Q1–Q3).

UICC T-status: group comparison was performed patients with T0–2 and those with T3–4.

UICC N-status: group comparison was performed patients with N0–1 and those with N2.

Stage: group comparison was performed patients with IA–IIB and those with III–IV.

### Cell culture

The human pancreatic adenocarcinoma cell line PANC-1 and MIAPaCa-2 were cultured in Dulbecco’s modified Eagle’s medium (DMEM) (SIGMA, MO, USA); BxPC-3, AsPC-1, KLM-1 and human acute monocytic leukemia cell line THP-1 were cultured in RPMI-1640 (SIGMA); Human pancreatic stellate cell line hPSC-1 were cultured in DMEM/F12 (Ham) (Gibco, MA, USA). PANC-1, MIAPaCa-2, AsPC-1, and THP-1 were obtained from the American Type Culture Collection (VA, USA), BxPC-3 was obtained from the European Collection of Authenticated Cell Cultures (Salisbury, UK). KLM-1 and hPSC-1 were from RIKEN BioResource Research Center (Ibaraki, Japan). All culture media were supplemented with 10% fetal bovine serum (FBS), penicillin (100 U/mL), and streptomycin (100 µg/mL), and cells were cultured in a 5% CO_2_ incubator at 37 °C. Cells with inducible single guide (sg)-RNA were maintained in DMEM supplemented with 10% Tet system approved FBS (Clontech Laboratories, Mountain View, CA, USA). Experiments were performed using cells within maximum of 15 passages after thawing.

### Reagents and antibodies

cGAS agonist, G3-YSD (Invivogen, CA, USA) was reconstituted with water and transfected to cells using TransIT-LT1 transfection reagent (Mirus Bio, WI, USA). The following antibodies were used for immunohistochemistry: cGAS (#79978, 1:50), STING (#13647, 1:100), CD206 (#24595, 1:500), FOXP3 (#98377, 1:100), and αSMA (#19245, 1:600) from Cell Signaling Technology (Danvers, MA, USA); CD8a (#ab101500, 1:100) from Abcam (Cambridge, MA, USA). The following antibodies were used for immunoblot analysis: cGAS (#79978, 1:1000), STING (#13647, 1:1000), phospho-IRF3 (#29047, 1:1000), phospho-TBK1 (#5483, 1:1000), IRF3 (#11904, 1:1000), TBK1 (#3504, 1:1000), GAPDH (#2118, 1:1000) from Cell Signaling Technology.

### Immunohistochemistry

All specimens were fixed in formalin and embedded in paraffin. Immunohistochemical staining was performed by an immune-peroxidase method. Tissue sections were blocked for 1 h at room temperature with 1.5% normal goat serum (Vector Laboratories, CA, USA) and subsequently incubated with primary antibody in 1.5% goat serum overnight at 4 °C. Bound antibody was detected using Simple stain MAX-PO (MULTI) peroxidase-conjugated secondary antibodies (Nichirei Bioscience, Tokyo, JAPAN) and diaminobenzidine (FUJIFILM Wako chemicals, Osaka, Japan). Sections were counter-stained with hematoxylin before mounting. The immunohistochemical grade was independently determined by two individuals or quantified using ImageJ software, version 1.51j8 (ImageJ, U.S. National Institutes of Health, MD, USA, https://imagej.nih.gov/ij/).

### Immunoblot analysis

Whole cell lysates were collected with RIPA lysis buffer (ThermoFisher, MA, USA). Proteins were resolved by 12.5% SDS-PAGE, transferred on to an Immobilon-FL PVDF membrane (EMD Millipore, MA, USA), and blotted with primary antibodies. Proteins were visualized using Clarity Western ECL Substrate (BIO-RAD, CA, USA) and ImageQuant LAS4000 imaging system (Cytiva, MA, USA). Blotting data was normalized to GAPDH by densitometric analysis using ImageJ software.

### RNA isolation and quantitative PCR

Total RNA was isolated from cells using TRIzol reagent (Invitrogen). NucloSpin total RNA FFPE kit (Macherey–Nagel, Duren, Germany) was used for isolating total RNA from formalin-fixed paraffin embedded (FFPE) tissues. Reverse transcription (RT) was performed using SuperScript RT reagent kit (TaKaRa Bio, Shiga, Japan). Quantitative PCR was performed using the TB Green Premix Ex Taq II kit (Takara Bio) on a StepOne real-time PCR system (Applied Biosystem, MA, USA). Target gene expression was calculated by the ∆∆Ct method and normalized to 18S ribosomal RNA expression levels. All primers used in this study were listed in Table 4.

**Table 4:** Primers used in this study.

Genes	Forward (5′ to 3′)	Reverse (5′ to 3′)
*18s*	CGCTTCCTTACCTGGTTGAT	GAGCGACCAAAGGAACCATA
*IFNB1*	CTTGGATTCCTACAAAGAAGCAGC	TCCTCCTTCTGGAACTGCTGCA
*IL6*	AGACAGCCACTCACCTCTTCAG	TTCTGCCAGTGCCTCTTTGCTG
*CD8a*	CTCCCAAAACAAGCCCAAG	AGGGTGAGGACGAAGGTGT
*CD4*	TGACTGCCAACTCTGACACC	GCACTGAGGGGCTACTACCA
*CD3*	ATTTTCGTCCTTGCTGTTGG	GGCTGGTAGAGCTGGTCATT
*CD19*	GTCTTATGGAAACCCGAGCA	ATAGCCCTCCCCTTCCTCTT
*CD11c*	CCCCCATTACTACGAGCAGA	GAACAGCATCACACCACCAC
*XCR1*	CAGCTAGAATACGCCCTGCT	ACTTGACCCCCACGAAGAC
*CD80*	TTGTGATATGCTGCCTGACC	GGGCGTACACTTTCCCTTCT
*CD86*	TGGAACCAACACAATGGAGAG	AACACGCTGGGCTTCATC
*CD206*	CGTTTACCAAATGGCTTCGT	GTACCCATCCTTGCCTTTCA
*CD163*	CCAGAAGGAACTTGTAGCCACAG	CAGGCACCAAGCGTTTTGAGCT
*CD56*	CCGTAGAAGCAAAGCCAGAG	TTTGTCTGTGTGGCGTCATT
*NKp46*	CAGTGAAGCTCCTGGTCACA	AAAAGGTAGGTGCCCCAAGT
*CD33*	CCTGCTCGCTCTTTGTCTCT	GTGGTAGGGTGGGTGTCATT
*CD124*	GCGATGTGTGGAGTTGTTTG	GAAGTCATCCCTGCTGCTCT
*FOXP3*	GCTGGAGAAGGAGAAGCTGA	GGATGATGCCACAGATGAAG
*αSMA*	CTATGCCTCTGGACGCACAACT	CAGATCCAGACGCATGATGGCA
*FAPα*	AGCTTCCTCGTCCAATTCAG	GGTGGATCTCCTGGTCTTTG
*TIMP-1*	CTGGCATCCTGTTGTTGCT	AGGTCGGAATTGCAGAAGG
*Meflin*	GACAGCAACGAGCTGACCTT	AGCGGTTGTGGTTGAGTTG
*STING*	GATAAACTGCCCCAGCAGAC	TGCCCACAGTAACCTCTTCC
*TNFA*	AGCCCATGTTGTAGCAAACC	GCTGGTTATCTCTCAGCTCCA
*IFNA*	GAATCTCTCCTTCCTCCTGTCTG	GCTGGAGCCTTCTGGAACT
*IFNB1 (for FFPE)*	TTGCTCTCCTGTTGTGCTTC	TCTGACACTGAAAATTGCTGCT
*IL6 (for FFPE)*	ATGCAATAACCACCCCTGAC	GCGCAGAATGAGATGAGTTG

### CRISPR/Cas9 genome editing

The sequences of sgRNA were designed by TrueDesign genome editor (ThermoFisher). They included a four-base pair (bp) overhang for the forward (ccgg) and complementary reverse (aaac) oligos to enable cloning into FgH1t plasmid (Addgene, MA, USA) as described previously^37^. To produce lentiviruses, FgH1t or FUCas9Cherry (Addgene) plasmids were transiently co-transfected into HEK293T cells with the packaging plasmids pMDLg/pRRE, pRSV-rev, and pCMV-VSV-G (all from Addgene) using the TransIT-LT1 transfection reagent (Mirus Bio). The sgRNA sequence for human *cGAS* is 5′-CGAACTTTCCCGCCTTAGGC-3′.

### Isolation of anti-tumor immune cells

Human healthy donor peripheral blood mononuclear cells (PBMCs) were isolated using Lymphocyte Separation Solution (Nacalai tesque, Kyoto, Japan). Human T cells and NK cells were enriched from PBMCs by cell depletion of B cells and monocytes using anti-CD19, and anti-CD14 conjugated magnetic beads with Magnisort technology (ThermoFisher). To facilitate visualization, purified T cells were stained with 2 µM Calcein-AM (Nacalai tesque) in PBS for 30 min at 37 °C followed by co-culture. Isolated cells were maintained in a T cell medium; RPMI1640 supplemented with 10% FBS, 100 U/mL human IL-2 and 10 ng/mL human IL-7 (both from Biolegend, CA, USA).

### Preparation of dendritic cells (DCs) and priming

Human monocytes were magnetically isolated using anti-CD19 microbeads. Isolated monocytes were cultured in RPMI medium supplemented with 10% FBS, human IL-4 and human GM-CSF (both from Biolegend) (as a DC differentiation medium) for 5 days. To allow maturation and vaccination of DCs, cells were further treated with conditioned media from BxPC3 cells for 24 h, followed by culturing with cell lysate of BxPC-3 cells. The next day, DCs were harvested and co-cultured with purified T + NK cells at a ratio of 1:20 (DCs:T cells) for priming.

### Immune cell migration/infiltration assay

Prior to co-culturing with T + NK cells, BxPC-3 cells were transfected with cGAS agonist in a 24-well plate (1 × 10^4^ cells/well). The next day, BxPC-3 cells were co-cultured with T + NK cells plated in transwells (pore size; 3.0 µm) (CORNING, CA, USA) with or without CAFs. After 24 h of co-culture, migrated or infiltrated cells were counted using Image J software. To evaluate cytotoxicity by T + NK cells, chemiluminescence cell viability assay was performed using CellTiter-Glo reagent (Promega, WI, USA). Chemiluminescence was measured using OPTIMA-6 multi-detection microplate reader (BMG Labtech, Ortenberg, Germany).

### Statistical analysis

Statistical analyses were performed using R software, version 4.0.3 (The R foundation, Vienna, Austria) or GraphPad Prism 9.3.0 (GraphPad software, La Jolla, CA, USA). Overall survival of patients was estimated using a Kaplan–Meier curve and compared using a log-rank test. Data are represented as mean ± standard error of the mean (SEM) from at least three independent experiments. To assess the statistical significance of differences, a two-tailed *t*-test (for two groups) or one-way analysis of variance (ANOVA) followed by Dunnett’s test or Tukey’s test (for multi groups) was performed.

## Supplementary Information

Supplementary Figure S1.

Supplementary Figure S2.

Supplementary Figure S3.
