# Supplementary material for: cGAS-STING signaling encourages immune cell overcoming of fibroblast barricades in pancreatic cancer
Source: Sci Rep. 2022 Jun 30;12:10466. doi: 10.1038/s41598-022-14297-5 (PMC9247053; doi:10.1038/s41598-022-14297-5)
Supplement: Supplementary file 3 — Supplementary Figure S3. [file 41598_2022_14297_MOESM3_ESM.docx]

**Supplemental Figure 3**

**a**

**b**


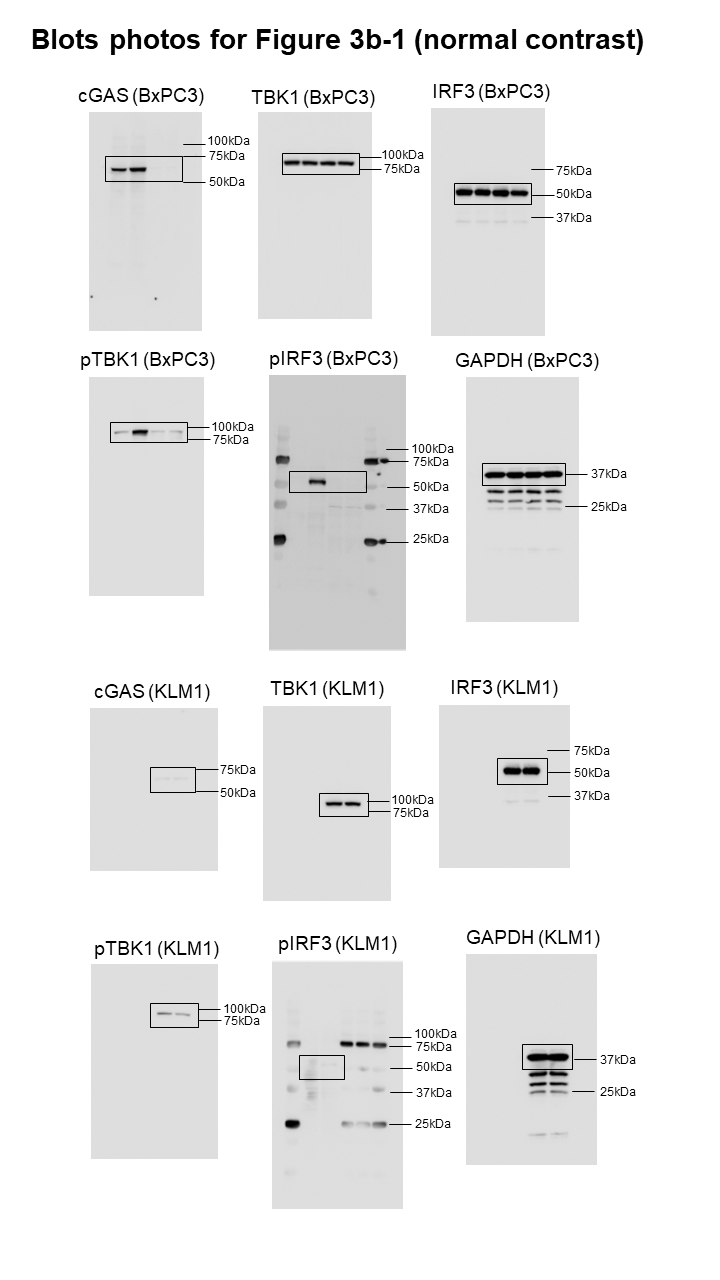

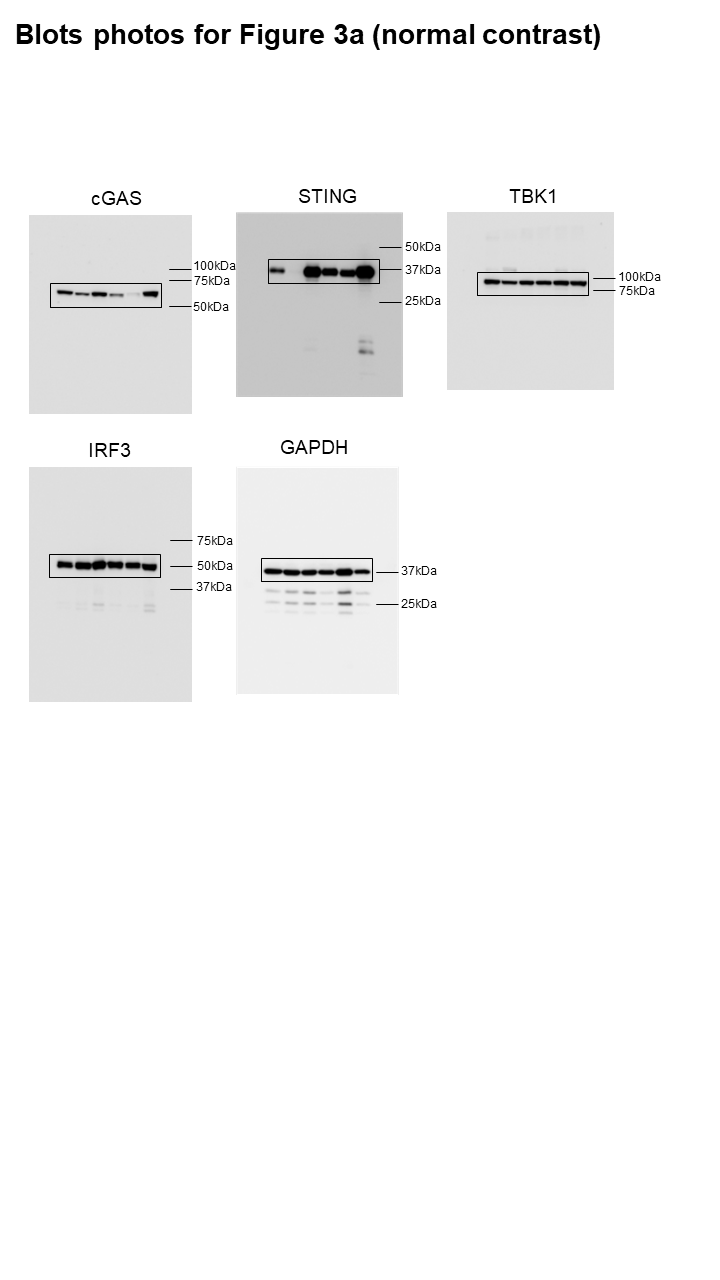


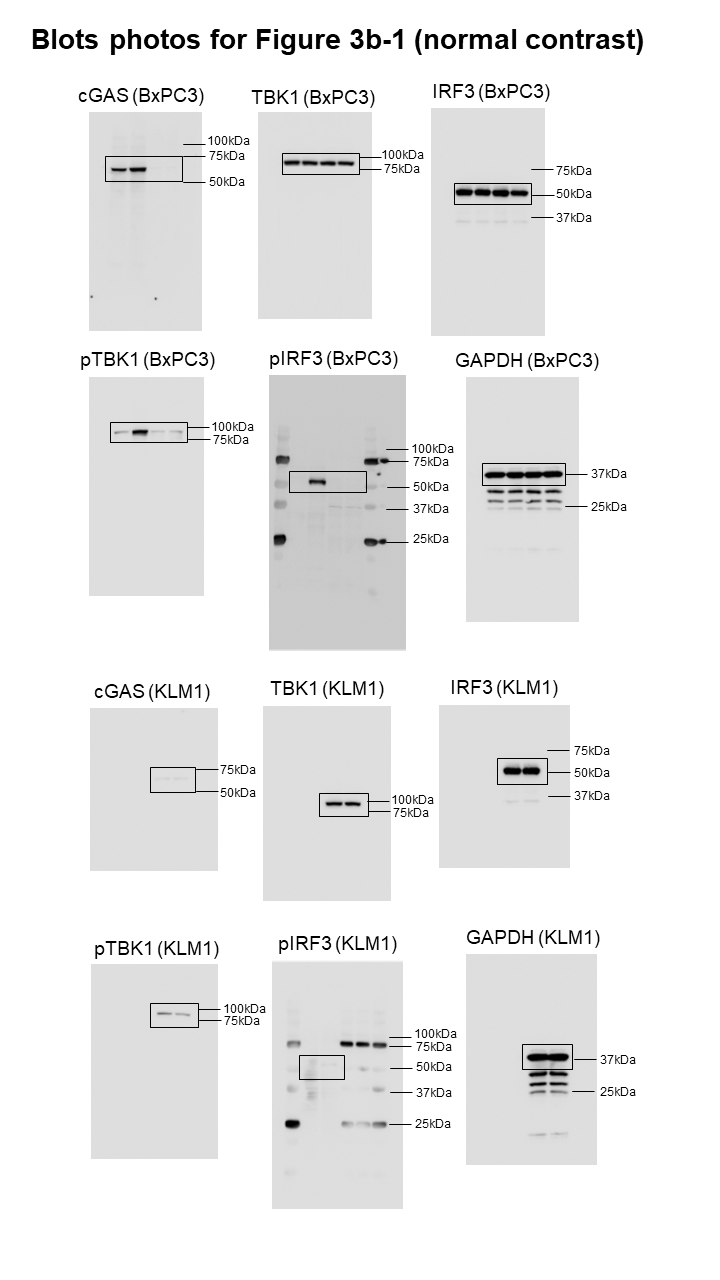


**c**

**d**

**e**

**
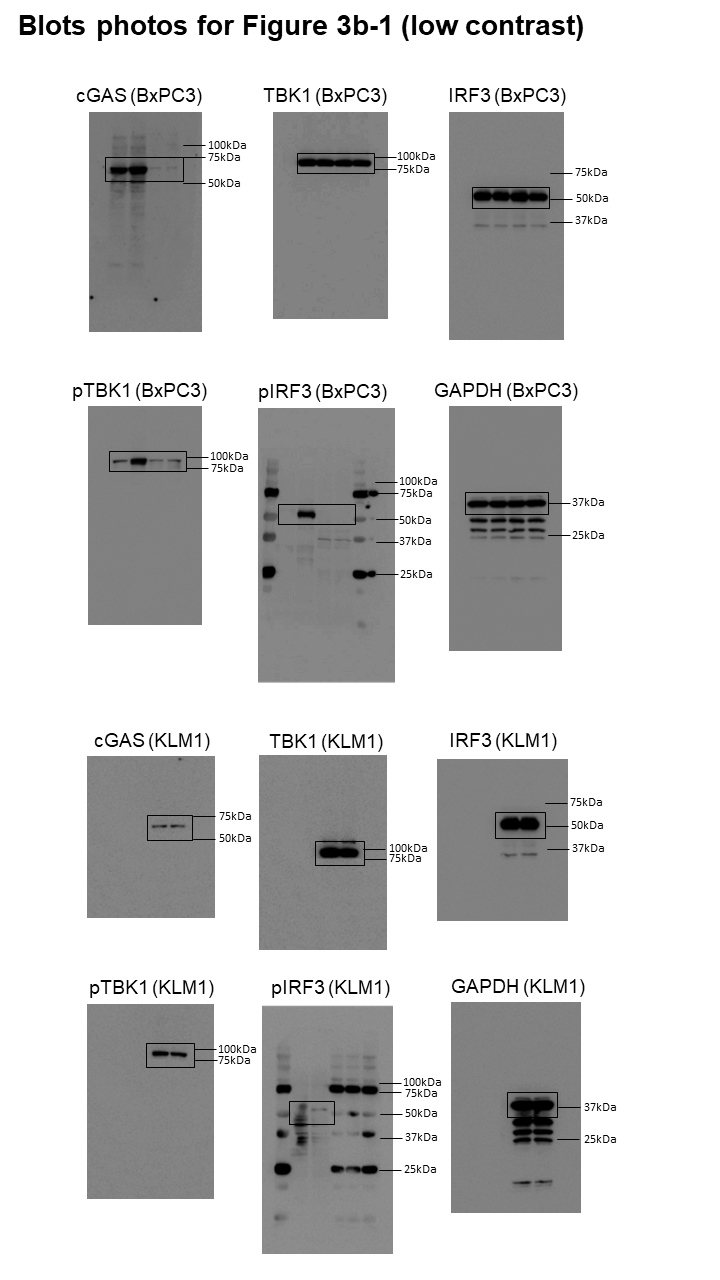
**


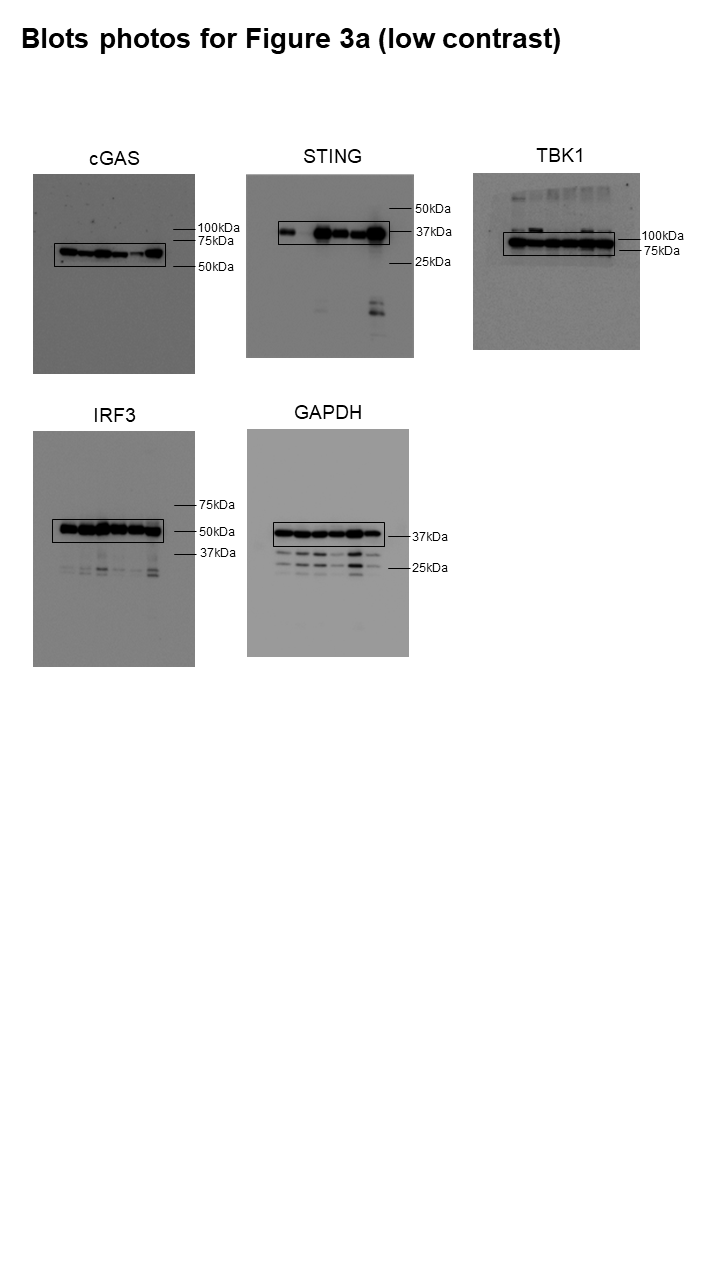


**f**

**
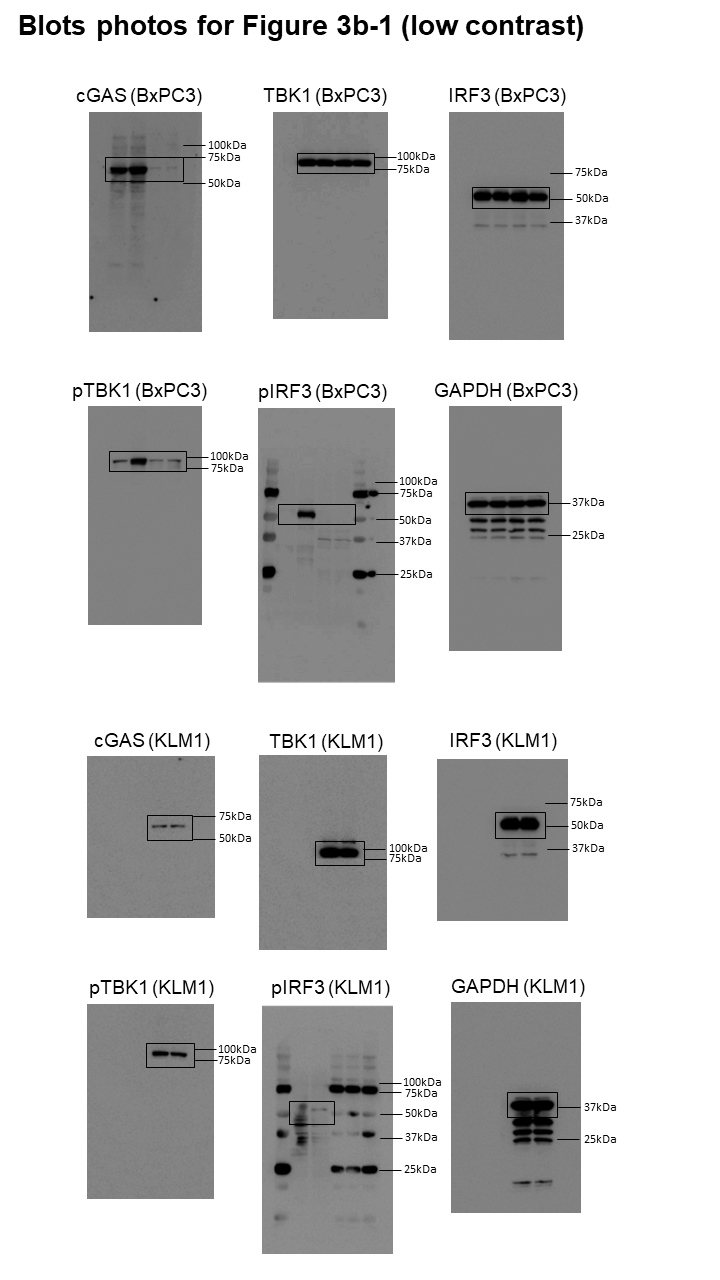
**

**Supplemental Figure 3.** **Original images of immunoblotting analysis.** (a) The original immunoblotting image of PDAC cell lines in Figure 3a. (b) BxPC-3 cells in Figure 3b. (c) KLM-1 cells in Figure 3b. (d) PDAC cell lines in Figure 3a (low contrast). (e) BxPC-3 cells in Figure 3b (low contrast). (f) KLM-1 cells in Figure 3b (low contrast).
